# Advances in Emerging Non-Destructive Technologies for Detecting Raw Egg Freshness: A Comprehensive Review

**DOI:** 10.3390/foods13223563

**Published:** 2024-11-07

**Authors:** Elsayed M. Atwa, Shaomin Xu, Ahmed K. Rashwan, Asem M. Abdelshafy, Gamal ElMasry, Salim Al-Rejaie, Haixiang Xu, Hongjian Lin, Jinming Pan

**Affiliations:** 1 College of Biosystems Engineering and Food Science, Zhejiang University, Hangzhou 310058, China; sayedrefaat@zju.edu.cn (E.M.A.);; 2 National Key Laboratory of Agricultural Equipment Technology, Zhejiang University, Hangzhou 310058, China; 3 Agricultural Engineering Research Institute, Agricultural Research Center, Giza 12618, Egypt; 4 Department of Food and Dairy Sciences, Faculty of Agriculture, South Valley University, Qena 83523, Egypt; 5 Department of Food Science and Technology, Faculty of Agriculture, Al-Azhar University—Assiut Branch, Assiut 71524, Egypt; 6 Department of Agricultural Engineering, Faculty of Agriculture, Suez Canal University, Ismailia 41522, Egypt; 7 Department of Pharmacology & Toxicology, College of Pharmacy, King Saud University, Riyadh 11451, Saudi Arabia

**Keywords:** egg, non-destructive technologies, freshness, NIR spectroscopy, hyperspectral imaging, computer vision

## Abstract

Eggs are a rich food source of proteins, fats, vitamins, minerals, and other nutrients. However, the egg industry faces some challenges such as microbial invasion due to environmental factors, leading to damage and reduced usability. Therefore, detecting the freshness of raw eggs using various technologies, including traditional and non-destructive methods, can overcome these challenges. As the traditional methods of assessing egg freshness are often subjective and time-consuming, modern non-destructive technologies, including near-infrared (NIR) spectroscopy, Raman spectroscopy, fluorescence spectroscopy, computer vision (color imaging), hyperspectral imaging, electronic noses, and nuclear magnetic resonance, have offered objective and rapid results to address these limitations. The current review summarizes and discusses the recent advances and developments in applying non-destructive technologies for detecting raw egg freshness. Some of these technologies such as NIR spectroscopy, computer vision, and hyperspectral imaging have achieved an accuracy of more than 96% in detecting egg freshness. Therefore, this review provides an overview of the current trends in the state-of-the-art non-destructive technologies recently utilized in detecting the freshness of raw eggs. This review can contribute significantly to the field of emerging technologies in this research track and pique the interests of both food scientists and industry professionals.

## 1. Introduction

Eggs are a frequently consumed food item that has played a significant role in human diets because of their high nutritional content and adaptability in the food industry. It contains high-quality proteins and other nutrients, including minerals, carbs, and easily digestible fats, and is consumed by a diverse spectrum of people worldwide. The amount of eggs produced globally by 2021 has exceeded 86 million tons. By 2030, estimations indicate that the overall global production will be 90 million tons of eggs [1,2]. Also, eggs are multifunctional ingredients and can simultaneously serve several technological functions in formulated food products due to their thickening, gelling, foaming, emulsifying, coloring, and aromatic properties [3]. Eggs in raw and processed forms are extremely important in food sectors due to their industrial applications in commercial food production manufacturers such as refrigerated liquid eggs, frozen eggs, dried eggs, and other egg products. Therefore, the overall quality of eggs in terms of freshness, safety, and microbiological aspects was always a point of consideration for researchers and producers [4]. As eggs’ quality cannot be improved after being laid, they should be well preserved and served without deteriorating parameters such as temperature, humidity, handling, storage, and aging during daily and commercial practices [5]. The loss of egg freshness due to aging causes physical and functional changes in the egg (e.g., thinning of the albumen and weakening of antimicrobial properties), but it only slightly affects the nutritional values of proteins, vitamins, and fats. The most notable changes are slight losses in some vitamins, bioavailability of some nutrients, and lipid quality [6,7]. There are tremendous changes that occur in eggs if they lose their freshness such as a loss of structural integrity, an increase in albumen and yolk pH, a decrease in Haugh unit score, a reduction in yolk index, and a decrease in viscosity. In addition, losing egg freshness leads to losing functional albumen properties and drastic negative effects on lysozymes, ovalbumin, and ovotransferrin [8]. As egg freshness declines, the antimicrobial functions of lysozyme and ovotransferrin are diminished, while ovalbumin undergoes structural changes that negatively affect its functional properties. These changes impact the safety, quality, and usability of the egg in both culinary and industrial applications [9].

Because the egg-laying season usually peaks in the summer, keeping eggs fresh during storage, shipping, and sales could be difficult. They can be easily degraded and become unsafe for ingestion when exposed to poor temperature and humidity conditions owing to microbial invasion. When a healthy chicken lays an egg, a sterile liquid is enclosed by a membrane that covers the eggshells, and an albumen layer that seals the stomata is secreted to protect the eggs from microorganisms. However, these protective mechanisms are temporary and susceptible to damage and microbial growth, resulting in a range of physical and chemical alterations in eggs, such as fat spoilage, protein decay, and sugar decomposition [10].

Egg freshness is one of the most important indicators for estimating an egg’s internal quality, which gradually decreases with egg aging [10,11]. Various international standards have been issued for eggs, including sensory properties [12], appearance, odor, and taste, as well as physical properties, such as the Haugh unit (HU) [13], air cell size [14], yolk index [15], and specific gravity. Chemical analyses, such as pH [16] and total volatile basic nitrogen (TVBN) content [10], are also used to determine egg freshness. These indicators can determine the freshness degree, shelf life, and suitability for different processing methods such as boiling, baking, and preservation. In China, egg freshness is classified into four grades: AA (super grade), A (first grade), B (second grade), and C (third grade). Europe has two classes for quality classification of egg freshness: class A, which is divided into two quality categories (extra fresh and category I), and class B. The US grades are AA, A, and B quality [10,17].

Traditionally, egg freshness testing has been performed through visual inspection and physical examination of the egg, involving cracking, opening, or smelling [10,18]. However, these methods are time-consuming and do not always provide an objective evaluation [10,18]. With technological advancements, non-destructive techniques for egg freshness assessment have emerged [19]. Non-destructive approaches use various imaging and sensing techniques to evaluate the internal and exterior quality parameters of an egg without damaging or altering it. These methods objectively assess egg freshness and other quality attributes, such as size, shape, and weight, which are beneficial for producers and sellers who require monitoring of egg quality. Combining traditional and non-destructive methods provides a comprehensive and accurate approach for egg freshness testing [20,21].

This review provides an overview of the current trends in non-destructive technologies for detecting the freshness of raw eggs. The advantages and the main characteristics of the non-destructive techniques as well as recent research developments in the field of egg freshness estimation are thoroughly presented. Although this topic has been recently reviewed [10,22], the current review is more comprehensive and it was built, designed, and formulated to provide the readers with all required information and knowledge about all non-destructive techniques in terms of their theory, fundamentals, and configurations first followed by their applications in egg freshness estimation. Also, this review highlighted the applications of cutting-edge modalities and modern technologies such as hyperspectral imaging, fluorescence spectroscopy, computer vision, and color imaging for egg freshness estimation. Moreover, the current article reviews the applications of electronic noses, electronic tongs, and nuclear magnetic resonance (NMR) in egg freshness assessment. As a result, our findings provide invaluable and complete insights into the emerging field of non-destructive technologies for detecting the freshness of raw eggs, sparking the attention of food scientists and industry experts.

## 2. Nutritional Compositions of Egg

**Figure 1:**
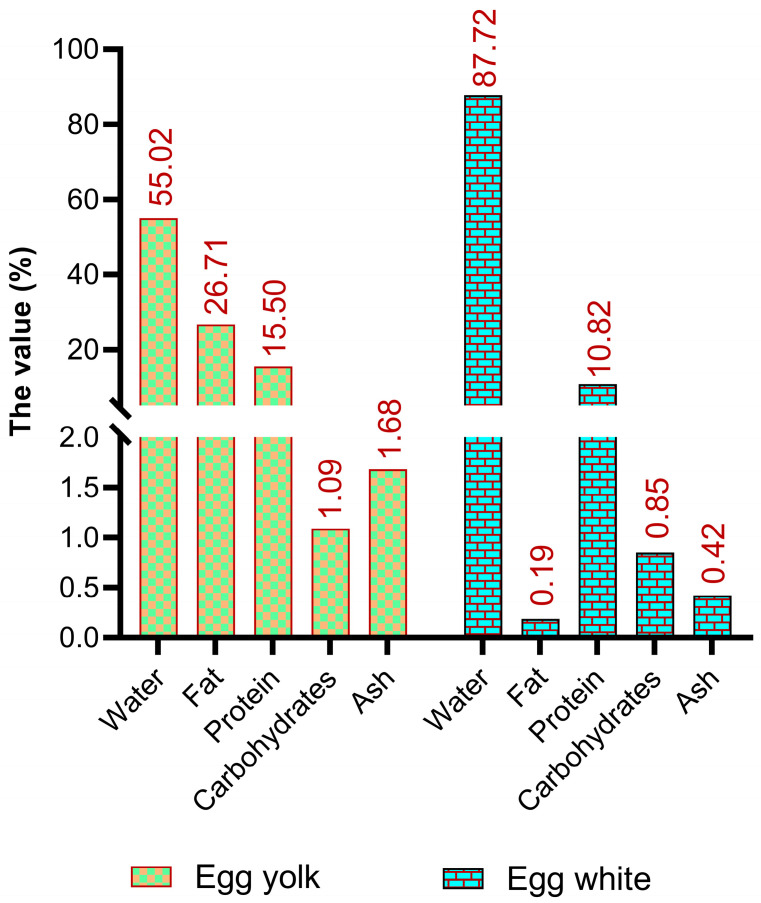
The basic composition of edible parts of an egg [24].

In addition to being an inexpensive source of protein and a modest calorie source (approximately 140 kcal/100 g), eggs contain a tremendous amount of essential fats, vitamins, and minerals (Figure 1) [23,24]. Although the relative concentrations of minerals, vitamins, and fatty acids in eggs differ among national standards, major components such as water, protein, lipids, and carbohydrates are globally comparable [24,25]. The stability of the primary nutrients in eggs primarily depends on the egg white-to-yolk ratio, whereas minor components can be affected by factors such as the hen’s diet. Fresh eggs typically contain approximately 76.1% water, 12.6% protein, 9.5% fat, 1.1% ash, and 0.7% carbohydrates [26].

Eggs contain various nutrients [27,28], including micronutrients such as choline, which have positive effects on brain health [29,30]. The frequency of egg consumption among children and women varies according to age, region, and socio-economic status. Also, cultural factors affect egg consumption during pregnancy and lactation; however, there are limited data on this topic. The implementation of social marketing and communication strategies has proven effective in elevating egg intake among children, while economic barriers are likely the main factor in low consumption [28,29]. An average egg contains 75 calories and is a rich source of high-quality protein, vitamins, minerals, carotenoids, and bioactive compounds. With 7 g of protein, 5 g of fat, and 1.6 g of saturated fat per egg, they provide a balanced and nutritious option. Additionally, the presence of lutein and zeaxanthin in eggs reduces the risk of age-related macular degeneration, a common cause of blindness in the elderly [28,31].

### 2.1. Macronutrients

Typically, the primary nutrients in food are referred to as macronutrients, which include proteins, fats, carbohydrates, fibers, and moisture. The protein content of egg white and egg yolk is considerably high. The primary source of egg yolk is the liver, whereas the egg white is produced and secreted in the hen’s oviduct following the release of the mature yolk during ovulation [32,33]. On average, a raw and uncooked whole egg contains 12.5 g of protein per 100 g, while the protein content of egg yolk and egg white is approximately 15.9 g and 10.90 g per 100 g, respectively [34,35]. Egg yolk is a complex mixture consisting of various components. The composition of egg yolk consists of approximately 68% LDL, 16% HDL, 10% soluble proteins such as livetins, and 4% phosvitins. These components are present in granules, which comprise 19–23% of dry matter, and in clear yellow plasma, which makes up 77–81% of dry matter and contains the remaining yolk proteins [36]. Yolk is strongly associated with vitelline membranes, which are composed of two distinct layers [23]. These membranes provide physical separation of the yolk from other egg compartments, preventing yolk leakage into the egg white. In contrast, the egg white is a gel-like structure that does not contain lipids and is mainly composed of water, comprising approximately 88% of its content. Egg white also contains ovomucins, a type of fibrous structural protein, glycoproteins such as ovalbumin and protease inhibitors, antibacterial proteins such as lysozymes, and various peptides [37]. A total of 150 different proteins have been identified in egg white, with ovalbumin being the most abundant, accounting for 50% of total egg white proteins [34]. Notably, egg white also contains four highly abundant protease inhibitors that impede the digestion of egg components, particularly when it is used as a raw ingredient in certain food preparations [23].

The lipid content in eggs remains relatively stable, ranging from 8.7 to 11.2 per 100 g of whole egg, and is concentrated mainly in the yolk [23,25], with a small portion remaining associated with the vitelline membranes [38]. The egg fat content varies with the yolk-to-egg white ratio. Moreover, the yolk is abundant in essential fatty acids such as linoleic acid (FA 18:2 9c,12c (n-6)). The previously held belief that the high cholesterol content of eggs (400 mg per 100 g of whole egg) contributes to cardiovascular disease led to a decrease in egg consumption a few decades ago [39]. However, studies in the 1990s reported no correlation between egg intake and high levels of plasmatic cholesterol and that egg consumption was not associated with a higher incidence of cardiovascular disease in healthy individuals, although those who are hyper-responsive to dietary cholesterol need to control their egg intake [23].

Eggs are devoid of fibers and have low carbohydrate content (0.7%). Eggs contain carbohydrates in both the yolk and white, with glucose being the primary free sugar. Glucose is more abundant in egg white, with around 0.34 g per 100 g of egg white compared to 0.18 g per 100 g of egg yolk. Other sugars such as fructose, lactose, maltose, and galactose are present in trace amounts in raw egg whites and yolk [23]. They contain a significant amount of carbohydrates, many of which are glycoproteins that undergo post-translational glycosylation before being secreted by the reproductive tissues of hens to form yolks, membranes, and egg whites.

### 2.2. Micronutrients

Micronutrients, referred to as minerals and vitamins, are essential components present in small quantities in foods. These vital substances are crucial for promoting healthy growth and development as well as preventing diseases. Previous studies have indicated that eggs are a rich source of micronutrients including vitamins, choline, minerals, and trace elements. Egg yolks are vitamin-rich sources, encompassing all vitamins except vitamin C (ascorbic acid) [23]. It contains high amounts of vitamins such as A, D, E, K, B1, B2, B5, B6, B9, and B12, while egg white is particularly rich in vitamins B2, B3, and B5 but also contains significant amounts of vitamins B1, B6, B8, B9, and B12 [40,41]. Consuming two eggs daily can meet 10–30% of the recommended daily intake of vitamins for humans. Furthermore, eggs contain a significant amount of choline, predominantly found in the yolk (680 mg/100 g in egg yolk compared to 1 mg/100 g in egg white) [30,42]. Among dietary sources, hard-boiled eggs are the second most abundant source of choline, following beef liver [43]. Furthermore, they represent the primary source of choline in the American diet [31]. Choline can be found in food in both water-soluble forms (such as free choline, phosphocholine, and glycerophosphocholine) and lipid-soluble forms (such as phosphatidylcholine and sphingomyelin) and plays a vital role in the cellular maintenance, growth, and development throughout all life stages. It has been linked to important bodily functions including neurotransmission, brain development, and bone health [44].

Eggs are abundant in phosphorus, calcium, and potassium and contain a moderate amount of sodium (142 mg per 100 g of whole egg) [23]. Furthermore, eggs contain all necessary trace elements, including magnesium, copper, iron, manganese, selenium, and zinc. Iron and zinc are mainly derived from egg yolk. The presence of certain minerals and micronutrients in eggs is noteworthy because a deficiency in some of them (e.g., Zn, Mg, and Se) has been linked to fatigue, depression, and the development of pathological illnesses [45].

### 2.3. Bioactive Compounds

Eggs are considered a great source of bioactive components, including phytosterols, phytoestrogens, tocopherols, carotenes, antioxidants, and n-3 fatty acids, which have been linked to various health benefits [46]. Eggs are a highly nutritious food with the potential to improve human health significantly [47]. They are rich sources of essential vitamins, minerals, and other nutrients that play crucial roles in various bodily functions. Additionally, eggs contain several biologically active compounds, such as antioxidants and omega-3 fatty acids, which have been linked to various health benefits. The combination of essential nutrients and bioactive compounds in eggs makes them an excellent food choice to promote overall health and well-being [23]. Whether consumed as a standalone food or as part of a balanced diet, eggs offer numerous health benefits that can affect different aspects of human health, from brain function and cardiovascular health to growth and development. Eggs are a nutritious food option, providing high-quality protein, vitamins, minerals, and choline for a relatively low-calorie count of 72 kilocalories per large egg [48]. Compared to other animal protein sources, eggs contain less saturated fat per gram, making them a healthier choice for those seeking to maintain a balanced diet. These essential nutrients and low-calorie count make eggs a great addition to any meal plan [49].

## 3. Detection Methods of Egg Freshness

Egg freshness is a crucial parameter that determines egg quality, safety, and shelf life [50]. With the increasing demand for high-quality eggs, there is a growing need for accurate and reliable methods for assessing egg freshness. Over the past 25 years, various detection methods have been employed on their physical, chemical, and biological properties. Two approaches have emerged to investigate egg freshness: traditional and non-destructive technologies. Traditional methods include sensory evaluation, HU measurement, and pH determination. Advanced technologies such as computer vision, low-field nuclear magnetic resonance, electronic nose, electronic tongues, dielectric spectroscopy, NIR spectroscopy, and hyperspectral imaging have recently been applied for the non-destructive detection of egg freshness [10].

### 3.1. Traditional Assessment Methods

#### 3.1.1. Sensory Evaluation

The sensory evaluation of egg freshness is a traditional method that involves human testing based on appearance, smell, and taste [10,51]. This method assesses various egg characteristics, such as shell appearance, egg white viscosity, yolk color, odor, and flavor. The sensory evaluation panel involves human experts using a standardized scoring system to rate the egg based on the presence of off-odors, off-flavors, and other factors. Sensory evaluation is a subjective method that relies on the personal judgment of testers. It varies depending on the testers’ individual preferences, experience, and sensitivity. However, it is still widely used in the egg industry as a reliable and cost-effective method to determine egg freshness. During a visual check, examiners detected the color and mold of the egg and the color of the albumen and yolk after being cracked out. Furthermore, the examiners relied on olfaction to detect the scent of egg liquid. Despite its simplicity, it requires a high level of skill from examiners, and the results are subject to bias or partiality [10]. Nevertheless, trained panelists possess the ability to discern variations in attributes like flavor, aroma, off-flavor, and overall characteristics in eggs sourced from hens fed with flax seeds. Karoui et al. evaluated the visual attributes of raw eggs, including the white, yolk, and whole egg, using a scale from 0 to 100. The study examined eggs stored at various temperatures (4, 18, and 32 °C) and durations (0, 7, 14, and 21 days), which revealed the strongest influence on temperature and, conversely, storage duration. Notably, panelists could distinguish significant differences between fresh egg yolks and those stored for 21 days at 18 °C. The study revealed a higher visual appeal of egg yolks stored for three weeks at 18 °C than those stored for seven days at 32 °C [52].

#### 3.1.2. Physicochemical Analysis

Egg freshness can be determined by performing various physicochemical measurements, including HU, yolk index, albumen pH, air chamber diameter, and air chamber height. The most common method for evaluating egg freshness is pH measurement because it is an important indicator of the acidity or basicity of food substrates. A fresh egg is a delicate food item that can deteriorate rapidly owing to bacterial contamination. Thus, the pH value provides valuable information about freshness. A fresh egg has a neutral pH value (7.6–7.9). pH value is increased gradually between 9.0 and 9.7 as carbon dioxide diffuses from the egg into the air through tiny pores called “stomata.” [53]. When eggs are stored for extended periods, there is a notable change in the chemical composition of the egg white, and the pH level of the egg white (albumen) increases, meaning that the egg white becomes more alkaline as it ages [54]. The correlation between albumen pH and HU is significant, and the pH level of albumen is generally not influenced by age or egg strain, indicating that storage duration and temperature significantly affect the shelf life of eggs [16]. Higher storage temperatures cause eggs to lose their freshness more quickly. Therefore, in regions with a humid tropical climate, it is not recommended to store eggs for more than three weeks at room temperature because the pH level of albumen increases to a level that is not typical of a fresh egg [55].

The height of the air cell in the egg can serve as a useful indicator of egg freshness [13]. The air cell inside it gradually becomes larger owing to moisture loss and gas exchange through the porous shell. Air cell results become taller and more prominent within the egg [56]. By examining the air cell size, egg freshness can be estimated using smaller air cells. The European Union regulation considers air cell height as the sole measurable parameter for determining egg freshness, which is dependent on egg weight. Egg grade A maintains an air cell height of less than 6 mm until the expiration date. However, because of the significant influence of temperature and humidity on the height of the air cell, it is challenging to ensure egg quality without proper regulation of these factors throughout the marketing process [52]. In China, there are strict guidelines for assessing egg freshness. According to these regulations, eggs are classified into four grades: super, first, second, and third. Each grade has a specific limit for the permissible air cell height, which is less than 4 mm, 6 mm, 8 mm, and 9.5 mm, respectively [57].

**Figure 2:**
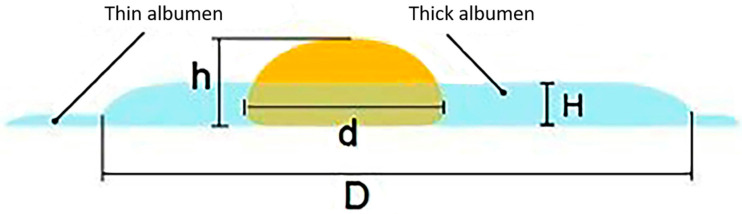
A scheme for measuring egg quality parameters of the thick egg white [59].

Egg white encompasses both the thick and thin types (Figure 2). The thick egg white gradually breaks down during storage, resulting in reduced quality. By comparing fresh and stale eggs broken onto a flat glass surface, it is evident that the thick egg white of fresh eggs adheres uniformly to the yolk. The thick egg white of stale eggs is scarce, with most of it being thin albumen. As a result, the thickness of the thick egg white can be measured using a vertical micrometer to evaluate egg freshness [10]. This method measures the egg white thickness (albumen), and the egg weight, and then calculates the HU using Equation (1). Its popularity has significantly increased over time.

(1) HU=100 log⁡ H−g30W0.37−100100+1.9      

Here, H is the height of the thick egg white in millimeters, W is the weight of the egg in grams, and g is the gravitational constant equivalent to 32.2 ft. s^−2^. Subsequently, Eisen et al. [58] simplified the previous equation by relieving a constant value and recalculating the entire equation, resulting in the following revised format of Equation (2):

(2) $$HU=100\;Log\left(H-1.7W^{0.37}+7.57\right)$$

The revised format of Equation (2) became the primary calculation method for determining the HU score. Additionally, Narushin et al. proposed an alternative index called the egg quality index (EQI) which is compared to the HU using simulation modeling [59]. The EQI considers the weight of the egg, in addition to the physical properties of the thick albumen and yolk, enabling a more precise categorization of eggs based on their quality grades. According to the findings, the EQI calculated using Equation (3) has broader potential for application. It includes an extra parameter that indicates yolk condition and results in a more precise grading of egg quality.

(3) $$EQI=100\log\left(\frac{100H\sqrt{H}}{\sqrt{0.5W-5+d^{2}H}}\right)$$

Here, d is the yolk diameter (mm).

According to Chinese regulations, the HU of eggs graded as AA quality must be over 72, while the HU of A-quality eggs must fall between 60 and 72. B-quality eggs must have an HU between 31 and 59, whereas eggs classified as C quality must have an HU below 31 [57]. In the US grading system, there are three grades: eggs graded as AA have an HU above 72, those graded as A have an HU between 60 and 72, and eggs graded as B have an HU below 60 [60].

Freshness can be estimated based on the measurement of product density. Two widely recognized methods for accurately and quickly determining the freshness of agricultural products based on density measurement are water displacement and flotation. However, these techniques are not practical for evaluating the freshness of some agricultural products, such as eggs, which tend to absorb water, as they are considered invasive or damaging and can skew the measurement results [61]. The freshness of eggs can be determined using the saltwater floatation method. This method involves the addition of 68 g of NaCl to 1000 mL of water, which is considered to be level 0. Each subsequent level, up to a total of 9 grades, is achieved by adding 4 g of NaCl. To determine an egg’s freshness, the egg is placed in the brine solution, starting from level 0, and the lowest brine specific gravity at which the egg floats is its freshness level [62].

Total volatile basic nitrogen (TVBN) is another traditional testing method used to assess egg freshness. TVBN refers to the total amount of nitrogenous compounds, such as ammonia and amines which have been converted to volatile substances through microbial activity or autolysis. As eggs age, the proteins in the egg yolk and white are broken down into volatile compounds, leading to an upsurge in TVBN levels. The TVBN content of an egg can be measured using various methods, such as distillation or titration, with higher levels indicating a lower level of freshness [63,64].

The theory behind the low yolk index method for testing egg freshness is based on the changes that occur in an egg as it ages. As an egg ages, the air cell inside the egg grows larger, and the egg white begins to break down and lose its viscosity. The yolk becomes more flattened and spreads out, and the egg white becomes thinner. This leads to a decrease in the ratio of yolk height to egg white thickness, which was measured to determine the yolk index. A lower yolk index value indicates that the egg is older and less fresh, and it can be calculated as shown in Equation (4) [65].

(4) $$Yolk\;index=\frac{Yolk\;high\;(mm)}{Yolk\;diameter\;\left(mm\right)}$$

A vertical micrometer quantifies the yolk height (mm), and a Vernier caliper measures the yolk diameter (mm).

For fresh eggs, the yolk index typically ranges from 0.30 to 0.50, with an average value of 0.42 [66,67]. However, it is important to note that the yolk index method is not always accurate in determining egg freshness because other factors can also influence the yolk index. For example, hen breed, hen age, hen diet, and egg storage conditions can affect the yolk index value [52,68]. Therefore, while the low yolk index theory is useful for understanding the changes that occur in an egg as it ages, it is not always a reliable indicator of egg freshness (Figure 2).

### 3.2. Non-Destructive Methods

Non-destructive testing methods that can be implemented without damaging the eggs are becoming increasingly popular for evaluating egg freshness. These methods allow for a non-invasive assessment of egg quality, making it possible to grade eggs while keeping them intact [69]. Some examples of non-destructive testing approaches for determining the freshness of the egg include near-infrared spectroscopy, Raman spectroscopy, fluorescence spectroscopy, computer vision (color imaging), hyperspectral imaging (HSI), electronic noses, and nuclear magnetic resonance (NMR). These methods can provide information not only on the morphological and physical quality features but also on the internal quality of the tested eggs without breaking them [10]. Other non-destructive methods include acoustic techniques, such as sound transmission and vibration analysis, gas sensing methods, and electronic tongues, which can detect volatile compounds released by the egg during storage. Non-destructive testing methods are a promising area of research in the field of egg quality assessment, as they can help reduce the number of wasted eggs during the sorting and grading processes and provide more accurate and reliable information for the egg industry. The current non-destructive testing methods for egg quality parameters are summarized in Table 1.

#### 3.2.1. Near-Infrared (NIR) Spectroscopy

**Figure 3:**
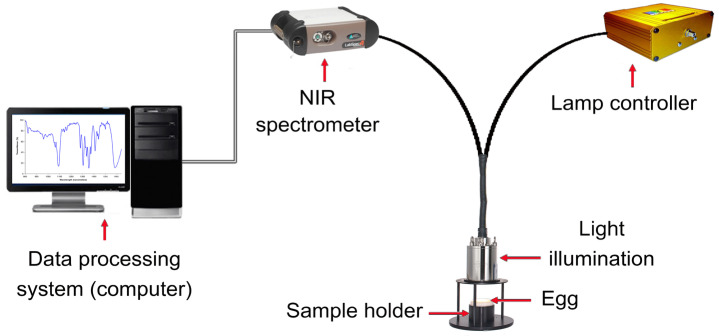
Schematic diagram of NIR spectroscopy components for non-destructive detection of egg quality.

Near-infrared (NIR) radiation occupies the electromagnetic spectrum segment, ranging from 780 to 2500 nm. NIR spectroscopy involves illuminating the product with NIR radiation and gauging the radiation, either reflected or transmitted. As this radiation interacts with the product, its spectral attributes change due to wavelength-dependent absorption and reflection phenomena. This alteration is contingent on the chemical composition of the product and its light-scattering traits, which are connected to its microstructure [94]. To decipher these intricate data, sophisticated multivariate statistical methods, like partial least squares regression (PLSR), are used to extract essential insights from the often intricate spectra. As shown in Figure 3, NIR spectroscopy comprises fundamental components, including a tungsten halogen light source, sample presentation accessory, monochromator, detector, and optical elements such as lenses, collimators, beam splitters, integrating spheres, and optical fibers [71,95].

NIR spectroscopy is a non-invasive technique for quickly assessing the physicochemical properties of eggs [73]. It relies on the interaction of near-infrared light with the sample, measuring the absorption of light caused by chemical bond vibrations, overtone bands, and combination bands [96]. The Beer–Lambert law determines the analyte concentrations in the examined samples [97]. Calibration models are first built using known samples to establish the relationship between spectral data and the constituents of interest [98]. These developed models are then used to estimate constituent concentrations. Consequently, NIR spectroscopy can be used to estimate egg freshness by detecting changes in chemical composition and physical attributes as eggs age [18]. This technique measures the absorbance or reflectance of light using samples that provide information about the molecular vibrations and composition of the egg in the near-infrared region of the electromagnetic spectrum [99].

The chemical composition of eggs, such as the degradation of proteins, lipids, and carbohydrates, changes over time. These changes can be detected and quantified using NIR spectroscopy [73]. By developing calibration models with known samples of varying freshness conditions, NIR spectra can be correlated with specific indicators of egg freshness, such as HU, yolk index, albumen pH, or other relevant parameters [100]. In the realm of egg freshness analysis, multivariate and chemometric models play a pivotal role in facilitating the classification and prediction of freshness levels. These models are categorized into two broad classes: supervised and unsupervised methods. Supervised models include techniques such as partial least squares discriminant analysis (PLS-DA), which draws boundaries between different freshness classes by identifying spectral variations related to freshness. Support vector machines (SVMs) are versatile tools that use spectral features to categorize eggs and ensure maximum separation between classes. Artificial neural networks (ANNs) are sought after for their capacity to capture intricate relationships in spectral data. In contrast, random forest (RF) employs an ensemble of decision trees to classify freshness levels, offering insights into feature importance [22]. On the unsupervised side, principal component analysis (PCA) reduces spectral data dimensionality and detects outliers. Hierarchical cluster analysis (HCA) helps group similar spectral patterns, providing insight into egg freshness relationships. K-means clustering segregates data into distinct clusters, revealing patterns in freshness, and self-organizing maps (SOMs) project high-dimensional spectral data onto a lower-dimensional grid to uncover underlying patterns. These models collectively contribute to the non-destructive evaluation of egg freshness, making informed classifications and predictions vital for the food industry [94].

For instance, NIR spectroscopy and pattern recognition approaches have been to differentiate between fresh and non-fresh eggs. Initial spectra of eggs in the range of 10,000–4000 cm^−1^ were collected; in addressing the challenge of an imbalanced number of training samples, a novel approach known as support vector data description (SVDD) was introduced to tackle the classification issue. The results of this study demonstrated that SVDD outperformed traditional classification models (i.e., PLS-DA, KNN, ANN, and SVM), with both fresh and non-fresh eggs identified at a rate of 93.3% [101]. Using a portable NIR spectroscopy (950–1600 nm) and chemometrics, egg samples were classified according to the production system (cage or free-range), based on the analysis of albumin and yolk spectra [102]. A portable NIR spectroscopy can be used to monitor the freshness of quail eggs during storage [103]. The data were modeled using optimized support vector regression to calculate the integrated freshness index (IFI) of brown-shell and pink-shell egg samples by analyzing their transmission spectra between 500 and 900 nm. The results showed that the model could perform non-destructive detection of both egg IFI variants simultaneously, indicating improved model reliability and wider applicability [71].

Syduzzaman et al. developed a technique using Vis-NIR spectroscopic (200–900 nm) and discriminant analysis (DA) to distinguish between double-yolked (DY) and single-yolked (SY) light-brown broiler eggs of similar size and shape via linear discriminant analysis (LDA) and support vector machine (SVM), achieving high accuracy rates of 96% and 100% in two separate experiments [72]. Tirado et al. reported that a small portable NIR spectrometer is a cost-effective alternative to benchtop devices for predicting egg freshness. Their findings indicate that the spectral range of 1300–1690 nm yields optimal results for predicting Haugh unit (HU) values and classifying egg freshness, achieving a relative error of 7.32% and a ratio of performance to deviation (RPD) of 2.56. Furthermore, the study demonstrated that partial least squares discriminant analysis (PLS-DA) outperformed support vector machine classification (SVM-C) in distinguishing between fresh and stale eggs, achieving an accuracy of 87.0% and exhibiting higher sensitivity in identifying stale eggs [73].

Quattrocchi et al. utilized near-infrared transmittance (NIRT) to assess egg freshness. The findings revealed that the partial least square (PLS) regression molding allowed the prediction of freshness variables such as days after hatching, air chamber size, weight loss, and pH, with correlation coefficients ranging between 0.9 and 0.92 [104]. However, it is essential to note that these high correlation coefficients apply to group averages rather than individual eggs. Karoui et al. reported on VIS-NIRT spectroscopy for egg quality assessment. The correlation coefficient between the spectral data of the examined egg samples and their reference pH values was 0.86, whereas for HU, it was 0.82 [52]. Wang et al. found that NIR diffuse transmission spectroscopy holds significant promise for distinguishing the storage duration of eggs under typical atmospheric conditions. The linear discriminant analysis model (LDA) correctly discriminated up to 91.4% of the samples in the prediction set. However, only 25.6% of the eggs stored under refrigerated conditions were correctly discriminated [70]. Due to its non-destructive nature, NIR spectroscopy preserves egg integrity, while providing a rapid and reliable assessment of freshness as demonstrated in Table 2.

#### 3.2.2. Raman Spectroscopy

**Figure 4:**
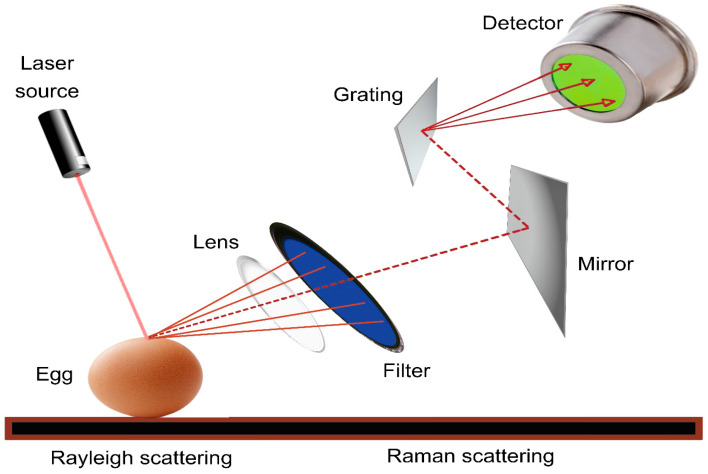
Schematic diagram of Raman spectral analysis for non-destructive detection of external and internal parameters of egg.

Raman spectroscopy is a method used to analyze the vibrational and rotational characteristics of molecules. Raman spectroscopy is primarily used to study the vibrational modes of molecules, and it can also provide information about rotational modes in certain cases, especially in gas-phase molecules. It works by detecting the inelastic scattering of photons by molecules, which provides insights into their vibrational and sometimes rotational energy levels [105]. It is a non-destructive analytical method that provides information on the chemical composition, molecular structure, and bonding of various materials [18,78,106,107]. The fundamental principle of Raman spectroscopy is based on the interaction of monochromatic light with a sample, resulting in the scattering of light at different wavelengths owing to molecular vibrations and rotations within the sample. In Raman spectroscopy, a laser beam is directed at the sample, and the scattered light is collected and analyzed. Most scattered light has the same energy (frequency) as the incident laser light, which is known as Rayleigh scattering. Nevertheless, a fraction of the dispersed light exhibits varied energies that align with the vibrational and rotational energy levels of the molecules in the sample. This shifted light is known as Raman scattering, and the energy shifts are characteristic of the molecular composition sample [18,78,106,107]. As shown in Figure 4, this instrument typically includes a laser light source, a monochromator to separate the scattered light, and a detector to measure the intensities of the scattered light at various wavelengths. The resulting Raman spectrum, which is a plot of intensity versus Raman shift, provides valuable information regarding the molecular structure and composition sample [22].

Liu et al. [19] developed a non-destructive and on-site method for assessing egg freshness using Raman spectroscopy and chemometrics. The researchers found a strong correlation between the Raman spectra of the eggshell surface and freshness, with correlation coefficients (R^2^) above 0.9 HU, albumen pH, and air chamber diameter, and above 0.8 for air chamber height using the partial least squares regression model (PLSR). This indicates that external eggshell information can be used to monitor egg freshness rapidly. Joshi et al. [77] conducted a study to assess the potential of Raman spectral analysis for the non-destructive and non-invasive identification of counterfeit eggs. Their findings demonstrated that Raman techniques, such as Raman spectroscopy and hyperspectral imaging, can effectively differentiate fake eggs from real eggs based on the distinctive Raman-sensitive properties of the chemicals used in fake egg production. This study identified critical wavebands within the spectral range of 1800–600 cm^−1^ that can be utilized to discriminate fake eggs from genuine ones accurately. As an efficient tool for monitoring and analyzing the vibrations of functional groups in eggs, the Raman spectroscopy technique has also been used successfully in analyzing eggs from different hens’ housing breeding systems as it is efficiently applied in estimating the quality of eggs based on typical spectra of lipids, proteins, and carotenoids [108]. Thus, Raman spectroscopy has been robustly employed for examining the shells and yolks to assess the quality and freshness of eggs and the results potentially indicate that the freshness changes led to Raman peaks alterations due to the Maillard reaction and oxidation of proteins and lipids and carotenoid depletion [109]. One of the most recent applications of Raman spectroscopy in estimating the overall quality and freshness of eggs was conducted by the same research team [78] that developed a technique for employing a scenario for using micro-Raman spectroscopy for examining both shells and yolks of eggs to assess their quality and freshness. The results emphasized that the technique could be efficiently used for detecting the freshness of the egg from its shell, indicating that the carotenoid Raman peaks have a noticeable effect on the determination of egg quality. Specifications and features of Raman spectroscopy are shown in Table 2 compared with the other non-destructive techniques.

#### 3.2.3. Dielectric Spectroscopy

**Figure 5:**
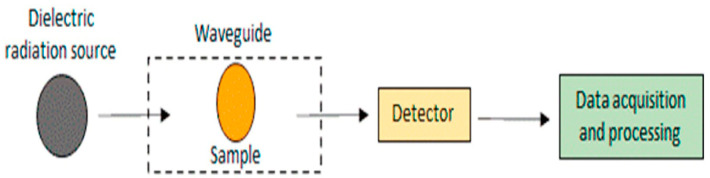
Block scheme of dielectric spectroscopy [22].

Dielectric spectroscopy is used to determine the electrical characteristics of materials and their frequencies (Figure 5) [22,110]. It is used to determine the freshness of eggs by measuring the dielectric properties of the egg white, which change over time as the egg ages [79]. As the egg white degrades and deteriorates, its electrical properties change, allowing the determination of egg freshness. By measuring these changes in the dielectric properties of the egg white, dielectric spectroscopy can assess the freshness of eggs non-destructively and quickly [111]. Quality assessment of the poultry eggs was performed using the dielectric technique. The dielectric technique measures egg samples at microwave frequencies and uses statistical or artificial intelligence models to process the dielectric spectra and quality indices of the eggs [80]. Soltani et al. used a rectangular waveguide, a sine wave sweep oscillator, and a spectrum analyzer to acquire measurements in the range of 3 to 20 GHz, with a 1 GHz increment and a time sweep of 60 s for each 1 GHz span. The authors utilized the dielectric detection technique in the radio frequency range for freshness detection using machine learning techniques such as artificial neural networks, support vector machines, decision trees, and Bayesian networks. The ANN with a topology of 62-18-6 achieved a flawless capability to predict the class of freshness with 100% accuracy, and different ML approaches were employed to estimate air cell height [79]. Lau and Subbiah developed an automated system that measures the temperature-dependent dielectric properties of egg components without human intervention. The dielectric constants generally decrease with frequency, and the temperature dependence of albumen and yolk is more complicated [112]. To achieve a more accurate and rapid determination of the egg yolk index, Sun et al. [113] investigated the correlation between dielectric properties and egg yolk index. Using these dielectric properties, they developed a non-destructive detection model for assessing egg freshness and obtained accurate egg yolk index information. The validation of their results showed a determination coefficient of 0.9115 and a yolk index error of ±4.2%.

#### 3.2.4. Fluorescence Spectroscopy

Fluorescence spectroscopy is a type of electromagnetic spectroscopy that analyzes fluorescence from a sample. It is a technique used to analyze fluorescence from molecules based on their fluorescent properties. The technique depends on detecting and identifying naturally occurring fluorescent compounds, known as fluorophores. The light emitted by these compounds arises when the fluorophores absorb energy, become excited at specific wavelengths, and then re-emit energy at different wavelengths. The intensity and wavelength of the emitted light are influenced by both the fluorophore itself and its surrounding chemical environment [114]. The amount of emitted energy and the wavelength at which the energy is emitted mainly depend on the fluorophore itself [22]. The key parameters such as excitation and emission wavelengths, Stokes shift, fluorescence lifetime, and quantum yield are critical for interpreting fluorescence data.

Fluorescence spectroscopy has witnessed considerable progress in being implemented as a rapid and non-invasive method for detecting concentrations up to one-thousandth lower than those measurable by standard absorption spectroscopy. It is a highly sensitive and rapid analytical method that detects fluorescent molecules such as tyrosine, phenylalanine, tryptophan in proteins, and vitamin A, providing insights within biological samples [82]. Many foods, particularly animal-derived products, contain numerous quality-related fluorescent groups, such as proteins, amino acids, and heterocyclic aromatic amines [115]. This makes fluorescence spectroscopy a valuable tool in food quality and safety studies. The technique has been applied to various products including cereals, oils, fruits, vegetables, and honey for quality analysis, variety identification, and adulteration detection [116]. It can also be used to monitor food spoilage by detecting specific fluorescent compounds, offering a reliable means to evaluate the freshness and safety of food products. Additionally, it plays a crucial role in identifying food adulterants and tracking compositional changes during food processing.

In egg freshness assessment, fluorescence spectroscopy was proven to be a reliable method for detecting egg quality based on estimating the presence of fluorescent molecules like tyrosine, phenylalanine, and tryptophan residues. The autofluorescence of fresh eggs is stronger than that of old ones since the intensity of autofluorescence depends on the amount of porphyrin on the shell surface. The lipid of egg yolk fat globules contains hundreds of triglyceride species. Fluorescence spectra were evaluated to discriminate eggs according to both their storage time and conditions [82].

Therefore, the technique has emerged as a promising technique for the non-destructive assessment of egg freshness, offering a potential alternative to traditional methods that are often time-consuming and subjective. However, the current body of research on this application is notably limited, with only a handful of studies exploring its efficacy in evaluating the overall quality of eggs and their freshness. For instance, Karoui et al. employed the fluorescence spectroscopy technique for monitoring egg freshness during storage based on the intrinsic fluorescence of thick and thin egg albumens at the fluorescence emission of tryptophan (excitation: 290 nm; emission: 305–430 nm) and fluorescence of Maillard reaction products (excitation: 360; emission: 380–580 nm). The data analyses using principal component analysis (PCA) and factorial discriminant analysis (FDA) indicated that tryptophan fluorescence spectra gave a classification accuracy of 62.5%; meanwhile, the fluorescent Maillard reaction products gave a correct classification accuracy of 91.4% for the validation set indicating that fluorescent Maillard reaction products could be considered as fingerprints that could be used for the discrimination between fresh and aged eggs. The same research group [117], Karoui et al., extended their study by using fluorescence spectroscopy to monitor egg freshness during storage based on fluorescence spectra of tryptophan and vitamin A. Using excitation at 360 nm, fresh thick albumen exhibited distinct fluorescence emission peaks at 410 and 440 nm, while aged eggs showed shifts in these peaks. The results revealed the high potential of vitamin A fluorescence spectra as useful fingerprints and effective tools for egg freshness, with fresh eggs showing the highest intensity at 410 nm and aged eggs showing a higher ratio of F.I.440 nm/F.I.410 nm [118]. Karoui et al. [82] further explored the use of vitamin A fluorescence spectra to monitor molecular changes in 225 eggs stored at 12.2 °C and 87% relative humidity under different CO_2_ conditions for 55 days. Vitamin A fluorescence provided clear discrimination of eggs based on storage time and conditions, distinguishing eggs aged 22 days or less from those stored 26 days or more. While overlapping occurred between certain age groups, such as eggs stored for 20 and 22 days or 26 and 29 days, fluorescence still proved as an effective method for identifying egg freshness in various conditions. Similarly, Karoui et al. employed the same front-face fluorescence spectroscopy routine for evaluating the freshness of a total of 126 intact brown-shelled eggs during different storage durations at a temperature of 12.2 °C and 87% relative humidity (RH) using emission fluorescence spectra of aromatic amino acids and nucleic acids (AAAs + NAs), fluorescent Maillard reaction products (FMRPs), and the excitation spectra of vitamin A. The best classification result of egg freshness (85.7%) was obtained when vitamin A fluorescence spectra (emission: 410 nm; excitation: 270–350 nm) were used as a powerful intrinsic probe for the evaluation of egg freshness [81].

**Figure 6:**
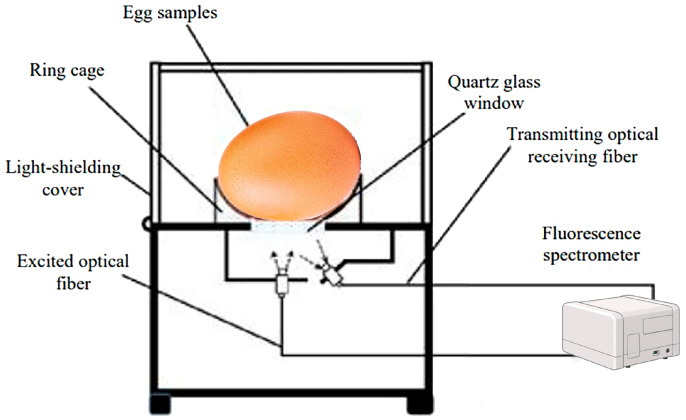
Direct measurement of the fluorescence spectra of egg samples with a laboratory fluorescence acquisition device [119].

In a study using synchronous fluorescence spectroscopy, the freshness of intact eggs was determined by analyzing fluorescence signals concentrated in two regions: A (excitation 290 nm, emission 320–380 nm) and B (excitation 380–570 nm, emission 610–735 nm). In the fluorescence spectroscopy technique, spectral information was obtained for excitation and emission by successively projecting a continuous excitation light wave onto a sample over a certain range of wavelengths, which can be used to obtain fluorescence information for various fluorescent groups at the same time. A multiple linear regression model based on optimal excitation–emission wavelength combinations was developed, achieving accurate predictions of egg freshness with an $R_{p}^{2}$ of 0.8879 and a root mean square error estimated by validation (SEP) of 6.2896, indicating the high potential of this technique for sensing the freshness of intact eggs (Figure 6) [119].

As eggs contain naturally fluorescent compounds, particularly in the yolk and albumen, such as riboflavin (vitamin B2), lipofuscin, and various proteins, the fluorescence properties of these compounds could be employed as key parameters to sense the egg ages due to alterations in their chemical structure or environment. The breakdown of proteins, the oxidation of lipids, and pH changes in the egg white all influence the fluorescence signals. As eggs age, the concentration of riboflavin decreases due to oxidative processes, and the pH of the egg white increases; these changes affect the fluorescence intensity of riboflavin, making it a useful marker for egg freshness. Also, proteins, particularly ovalbumin, undergo structural modifications during storage, including denaturation and aggregation. These changes affect their intrinsic fluorescence properties, primarily related to aromatic amino acids like tryptophan. The fluorescence intensity of these proteins decreases with age due to the alteration in their tertiary structure. Accordingly, the fluorescence intensity in the UV–visible range could be linked to the degradation of egg proteins and the oxidation of lipids, which are markers of egg aging. As eggshells emit vivid red autofluorescence by ultraviolet radiation, due to the presence of porphyrin on it, the autofluorescence of a fresh egg is stronger than that of an aged one because the intensity of autofluorescence depends on the amount of porphyrin on the shell surface. Consequently, fluorescence spectroscopy could be a promising approach for the quantitative estimation of porphyrin in eggs and thus for the determination of egg freshness [52,120].

Some other studies have demonstrated the method’s potential to detect changes in egg quality, but the existing findings require further validation and expansion. Given the technique’s ability to provide rapid and objective measurements, there is a clear need for more comprehensive research to fully understand its capabilities and limitations in the context of egg freshness detection. This will not only enhance our understanding of the underlying principles but also pave the way for the development of practical tools for the egg industry.

#### 3.2.5. Computer Vision/Traditional Color Imaging

**Figure 7:**
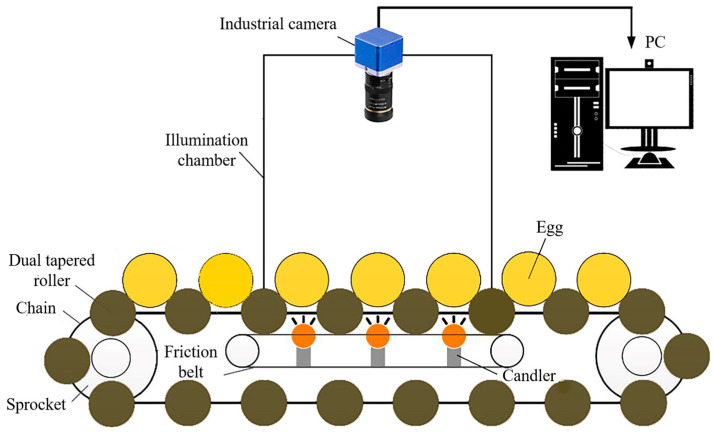
Egg image acquiring system based on machine vision. Source: the authors in the study by Guanjun et al. [85].

The primary component of a computer vision system is its high-resolution camera, which captures detailed images of eggs, enabling precise measurements of parameters such as shape, size, and surface irregularities (Figure 7) [61]. These images are processed using advanced image-processing algorithms to extract relevant features. The system’s effectiveness is enhanced by an illumination system that provides optimal lighting conditions, as image quality is highly dependent on lighting performance [10]. Additionally, the integration of color imaging enriches visual data processing, improving applications in object recognition and quality control. This capability allows for more nuanced decision-making and innovation across various industries. The digitization process converts visual images into numerical data, with software frameworks like MATLAB, ImageJ, and OpenCV playing a crucial role in image manipulation and analysis [121].

Computer vision technology has become a common tool in egg freshness evaluation, offering a non-invasive and efficient alternative to traditional testing methods. The eggshells allow for transparency, and the size of the air cell and ellipticity of the yolk can be seen using transmission light. Predictive models for freshness indices using machine vision have been established based on these observations [122,123]. Harnsoongnoen and Jaroensuk proposed a new approach for grading and evaluating eggs based on their freshness in a non-invasive, non-destructive, low-cost, straightforward, and real-time manner, using machine vision and a weight sensor based on density detection. They developed a machine vision system to assess the external physical characteristics (length, breadth, and volume) of the examined eggs. Additionally, they developed a weighing system to measure egg weight. Using egg weight and volume, they calculated density for grading and freshness assessment. The proposed system achieved impressive accuracy levels of 99.88, 98.26, and 99.02% for the weight, volume, and density measurements, respectively. Computer vision technology enables real-time and comprehensive analysis of egg freshness using classification model [61]. A device that integrates machine vision with dielectric constant detection was developed by Soltani et al., who established a model for estimating egg freshness through multi-source data fusion. This study examined the quality factors of eggs, including yolk index, HU, yolk/albumen ratio, and yolk weight. During the validation of the artificial neural network (ANN), high coefficient of determination (R^2^) values were obtained: 0.998 for HU, 0.998 for yolk index, 0.998 for yolk/albumen ratio, and 0.994 for yolk weight [79].

A machine vision system was used to estimate the mass of fresh eggs. They used six algorithms designed for the extraction of egg characteristics, including measures such as the minimum and maximum radius, effective radius, front area, and perimeter from each image. By using two perpendicular views of each egg, the system was able to accurately predict the egg mass with a correlation coefficient of approximately 95%. The best model was found to have predictors of area and effective radius [83]. A multiple linear regression (MLR) model was created to predict the HU using egg weight, long axis, and minor axis parameters, achieving good performance with an R^2^ of 0.8653 and a root mean square error of prediction (RMSEP) of 3.7454 in the prediction set [84]. The development of a computer vision system is aimed at monitoring egg freshness during storage. The Levenberg–Marquardt algorithm was used and proved to be the best in terms of predictive performance for the HU and albumen pH, with a correlation coefficient of 0.93 and 0.87, respectively [13]. Computer vision analysis of transillumination images of egg yolk and air cells obtained using a reverse-direction lighting method can accurately predict the freshness and storage time of eggs [124]. Guanjun et al. [85] introduced a novel machine vision approach for crack identification in eggs. Their experiments demonstrated the effectiveness of this method even in cases with challenging egg surface conditions, including irregular dark spots and microscopic cracks. The recognition rate of cracked eggs was 92.5%. A machine vision system for detecting egg cracks utilizing a modified pressure chamber and an egg that rotates continuously was designed and implemented by Priyadumkol et al. [86]. They created a reliable algorithm for crack detection and feature selection refinement. Their experiments, based on 750 egg surface images, achieved a 94% accuracy rate with only 1.67% false negatives. In conclusion, the use of computer vision techniques, such as spectroscopy, image analysis, and artificial neural networks, has shown great potential for estimating the internal and external freshness and quality of eggs, offering a non-destructive and efficient approach for the egg industry [86].

#### 3.2.6. Hyperspectral Imaging

**Figure 8:**
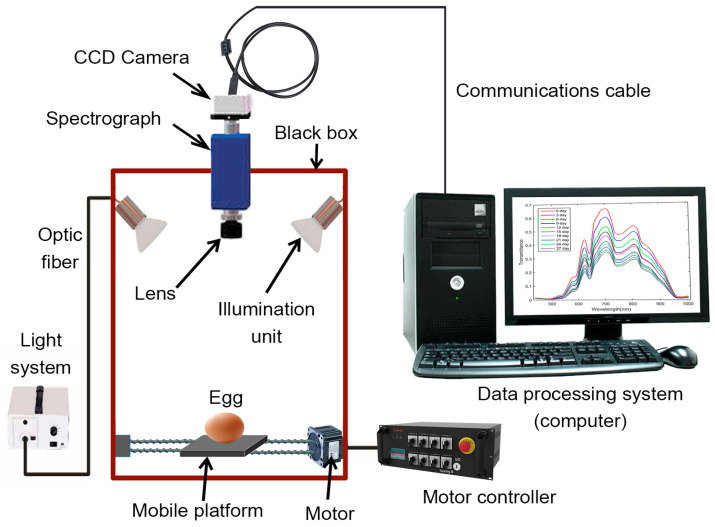
Schematic diagram of hyperspectral imaging technology as non-destructive testing of egg quality.

Hyperspectral imaging (HSI) technology is a new non-destructive technique that acquires extensive spatial image information of samples at different wavebands and captures spectral details for each pixel in the image (Table 2) [125]. The HSI technique generates numerous images of the same material across neighboring wavelength bands, typically with intervals smaller than 10 nm. These images, taken at different wavelengths, collectively comprise a hypercube (X, Y, λ), a three-dimensional dataset comprising a spectral dimension (wavelength λ axis) and two spatial dimensions (X- and Y-axes). The spatial dimension indicates pixel locations, whereas the spectral dimension illustrates the spectral data for each pixel. Two methods can be applied to analyze this hypercube. In the first approach, a spatial image (X, Y) is extracted from the hypercube at a specific wavelength (λ). This extracted image reveals variations in the spectral reflectance of the imaged samples at that wavelength, corresponding to differences in the distribution of their chemical components. In the second approach, we can extract the spectrum R (λ) from a particular pixel (X, Y). The extracted spectrum serves as a unique spectral fingerprint for each pixel, describing the chemical composition of the sample [126]. Hyperspectral image acquisition can be achieved using three main methods: point scanning (whiskbroom), line scanning (pushbroom), and area scanning (wavelength scanning). Area scanning, distinct from previous methods, acquires an image of the sample at a single wavelength during each scan and repeats this process across all wavelengths. Finally, the images at the corresponding wavelengths are stacked to produce a hyperspectral image. Furthermore, a typical HSI system comprises hardware and software components. The hardware includes essential elements such as a light source (typically halogen lamps), optical fibers for light transmission, a spectrograph to disperse light into different wavelengths, a CCD camera for signal conversion, a lens for focal adjustments, a movable stage for sample transport, and a controlling computer (Figure 8). On the software side, all functions are controlled by the computer, which includes software for managing the movable stage and acquiring hyperspectral images. Line scanning is the most commonly used method in agricultural product quality analysis because of its compatibility with food-processing conveyor belt systems [127].

Hyperspectral imaging technology is a non-destructive technique that acquires extensive spatial image information of samples at different wavebands and captures spectral details for each pixel in the image [125]. Thus, it provides both spectral and spatial information regarding the examined samples. Presently, hyperspectral technology has various applications in the non-destructive testing of grains, fruits, and vegetables [128]. However, limited research has been conducted on its use for evaluating egg freshness. Among such studies, utilizing reflectance hyperspectral imaging for detecting egg freshness offers quick and non-destructive grading with a correlation coefficient of 0.93 [129]. Furthermore, employing the optimal classification model iteratively retains informative variables, and the genetic algorithm support vector machine (IRIV GA-SVM) results in a classification accuracy of 99.25% for the training set and 97.87% for the test set [130].

Dai et al. introduced a technique based on hyperspectral scattering imaging to enhance the accuracy of egg freshness expressed by the HU. Their study involved a comparison of egg freshness prediction utilizing six distinct models: support vector machine (SVM), k-nearest neighbor (KNN), random forest (RF), naive Bayes (NB), discriminant analysis classifier (DAC), and latent Dirichlet allocation (LDA). The results revealed that the scattering hyperspectral method achieved the highest accuracy, with percentages of 100.00% for scattering, 88.75% for reflection, 95.00% for transmission, and 96.25% for mixed hyperspectral methods, signifying its superiority in egg freshness detection compared with other methods [88]. A novel approach for identifying and monitoring egg quality is proposed by utilizing hyperspectral image data of egg yolk and its spectral characteristics. This method offers an improved and precise egg classification. These findings demonstrate its effectiveness in detecting egg freshness and provide valuable insights and references for egg quality classification and detection [131].

In another study, Suktanarak et al. explored the application of reflectance near-infrared hyperspectral imaging in the spectral range of 900–1700 nm to non-destructively predict egg freshness expressed as HU. Their proposed method achieved a coefficient of determination (R^2^) of 0.91 and a root mean square error of calibration (RMSEC) of 4.58. The results indicate the potential of near-infrared hyperspectral imaging for generating egg images correlated with HU, enabling the non-destructive evaluation of hen egg freshness [87]. Zhang et al. assessed critical factors related to internal egg quality by creating a non-destructive testing method using hyperspectral imaging within the 380–1010 nm range. This method allows them to evaluate aspects such as freshness, bubble formation, and scattered yolk. They achieved an R^2^ coefficient of determination of 0.87 for freshness, while their system exhibited the capability to distinguish eggs with internal bubbles with 90% accuracy and those with scattered yolk with 96% accuracy. A freshness detection model was developed by combining the successive projection algorithm (SPA) with support vector regression [89].

#### 3.2.7. Electronic Noses and Tongues

E-noses and E-tongues are innovative devices that simulate the human senses of smell and taste to assess the freshness, aroma, and flavor of food [132,133] including eggs [90]. These technologies measure sensory quality by detecting differences in smell profiles and taste parameters, such as bitterness and astringency [134]. The results from E-noses and E-tongues provide crucial information about the quality and safety of egg products, enabling accurate discrimination between different egg breeds based on their sensory properties. This capability is essential for ensuring that consumers receive high-quality, fresh eggs.

Dong et al. compared the textures, odors, and tastes of eggs from three different breeds, applying LDA, fine k-nearest neighbor (KNN), and LSVM to achieve a perfect classification accuracy of 100% using E-nose data. When analyzing electronic tongue data, LDA and fine KNN trees attained the highest classification accuracy of 96.7% for yolks, while LDA reached 88.9% for albumen, and fine KNN achieved 87.5% for whole eggs. These findings highlighted variations in the smell profiles of the albumen and yolks as detected by the E-nose, whereas the E-tongue revealed differences in taste profiles [90]. Moreover, the use of fast gas chromatography (GC) with E-noses effectively determines the freshness of eggs based on different storage times through principal component analysis (PCA) and discriminant factor analysis (DFA). The accuracy of it in predicting storage time, Haugh units (HUs), and sensory scores was validated, showing high correlation coefficients (R^2^ = 0.9441 to R^2^ = 0.9725) for various quality metrics. This establishes E-noses as a reliable tool for monitoring egg freshness. In addition, Li et al. applied wavelet energy analysis to E-nose data, correlating storage time with the yolk index [91]. Four sensors in a quartz crystal microbalance (QCM) sensor array were used to evaluate the shelf life of eggs by modifying the surface of each sensor with different sensitive materials, which improved the cross-sensitivity and correlation of the array [93]. Liu et al. demonstrated that an E-nose could effectively predict the total volatile basic nitrogen (TVB-N) content of eggs using a support vector regression (SVR) model [135]. Furthermore, Gao et al. utilized the E-tongue and UHPLC-MS/MS techniques to analyze preserved egg yolk, identifying umami as the main taste and key umami substances, including glutamic acid and nucleotides [136], while Xiang et al. characterized the volatile organic compounds (VOCs) emitted from unfertilized, infertile, and fertilized eggs using SPME-GC-MS combined with E-noses, exploring potential biological information or fertilization-specific VOCs [137].

The adoption of E-nose technology as a non-destructive method for detecting egg freshness shows great promise for improving quality control and efficiency in the egg industry. By analyzing the volatile compounds emitted by eggs at various freshness stages, E-noses provide a rapid and objective assessment. In contrast, E-tongues, while modern tools for evaluating taste and quality, are not considered non-destructive due to their reliance on direct contact with the samples. Challenges remain, such as the need for extensive datasets to refine detection algorithms and the cost and maintenance of E-nose devices. However, with ongoing research and development, E-nose technology is well positioned to transform the assessment of egg freshness in the future. Specifications and features of the E-nose are shown in Table 2.

#### 3.2.8. Low-Field Nuclear Magnetic Resonance

Low-field nuclear magnetic resonance (LF-NMR) is a non-destructive technique that utilizes the magnetic properties of atomic nuclei to measure the physical and chemical properties of materials [138,139]. The use of LF-NMR and magnetic resonance imaging (MRI) has been recognized as an effective approach for studying the dynamics of water and lipid molecules in food [140]. These techniques are attractive because of their rapid analysis speed, non-invasiveness, and ability to provide real-time monitoring of spatial water or fat dynamics, making them potential tools for processing and quality control in the food industry [141]. LF-NMR and MRI have shown promising results in studying the behavior of water and lipid molecules in food and have the potential to become invaluable tools for monitoring the quality and freshness of food products [142]. In the context of egg freshness, LF-NMR is used to measure the transverse relaxation time (T2) of protons in egg components, such as the yolk and albumen. T2 reflects the mobility of water molecules in these components, which changes as the egg ages and deteriorates [143]. By analyzing the T2 values of different egg components, the freshness and quality of eggs can be determined. LF-NMR is a rapid, accurate, and non-destructive method for assessing egg freshness, as well as for detecting other quality attributes such as eggshell strength and yolk viscosity [144]. The application of LF-NMR technology is prevalent in the food industry for assessing quality through the analysis of relaxation characteristics and MRI of various components such as water, protein, or oil present in food [145,146,147]. Moreover, Laghi et al. [148] used MRI to investigate egg freshness by analyzing relaxation signals and observed changes in the 1H proton spin environment caused by increased albumen pH values that were related to egg freshness. The limitations of their study were the lack of inversion spectra calculations, the short storage period of eggs, and the low temporal resolution of the MRI instrument, which made it difficult to observe the protein hydrolysis process related to egg freshness. Hills et al. [149] performed a quantitative analysis of proton relaxation using NMR in both the egg albumen and yolk. Their findings showed that a 2D T1-T2 spectrum could distinguish between exchangeable and non-exchangeable proton pools, as well as the lipid proton pool in the egg. LF-NMR has shown promise as a non-destructive and rapid tool for estimating the internal and external freshness and quality of eggs, based on the analysis of water and lipid dynamics in egg components. The ability of LF-NMR to detect changes in the molecular environment of egg components offers an avenue to monitor the freshness and quality of eggs during storage and transportation. It has the potential to be a valuable tool in the egg industry (Table 2).

## 4. Challenges and Future Trends

In recent years, the rapid development of Raman spectroscopy technology has been recorded owing to notable progress in instrumentation. This technique shows some advantages over the NIR spectroscopy technique such as providing narrow spectra with sharply resolved bands, being insensitive to the sample’s physical form, and suffering from much less water interference [150]. However, Raman spectroscopy usually requires a longer acquisition time and a more expensive instrument compared with NIR spectroscopy. Therefore, more investigations are required to deepen the potential of Raman spectroscopy in the field of eggs and to advance cheap miniaturized instruments, ensuring the acceptable quality of acquired spectra at affordable costs and in a short time [22,151]. Dielectric spectroscopy is a recent non-destructive technique used for the examination of food quality and has attracted great attention in recent decades [152]. More studies should be conducted for better-oriented conclusions even if the few investigations carried out with dielectric spectroscopy in the field of shell eggs exhibited promising results regarding accuracy. Using non-destructive spectroscopic approaches, not all parameters of egg quality have been examined such as shell strength. Thus, much work is still required to widen possible applications. The solution-oriented spectrometers, offering rapid, easy, and cost-effective measurements, are possibly the next future of spectroscopy applications for non-destructive evaluation of shell egg freshness and quality, in view of both industrial online equipment and portable devices.

## 5. Conclusions and Future Directions

Eggs are excellent high-quality protein sources containing all essential amino acids and other nutrients required in our diets. The concept of freshness is central to assessing egg quality, which gradually declines with egg age. International standards employ various sensory and physical parameters, including appearance, odor, taste, and measures like the HU, air cell size, yolk index, and specific gravity, to gauge freshness. Additionally, chemical analyses such as pH and total volatile basic nitrogen content were performed for this purpose. Traditionally, the evaluation of egg freshness relies on visual and physical inspection methods, which, though informative, are time-consuming and subject to variability. However, recent advancements in non-destructive technologies have revolutionized this process, offering rapid and objective evaluations. Techniques such as near-infrared spectroscopy, Raman spectroscopy, fluorescence spectroscopy, electronic noses, computer vision, magnetic resonance imaging, and hyperspectral imaging have provided detailed insights into egg quality without the need for physical alteration. Notably, NIR spectroscopy, computer vision, and hyperspectral imaging have demonstrated exceptional accuracy (exceeding 96%), in assessing egg freshness. By harnessing these non-destructive methods, researchers and industry professionals can obtain precise information on both the external and internal quality of eggs, enabling informed decisions regarding their suitability for various processing methods and shelf-life estimations. The integration of advanced technologies into egg quality assessment underscores their invaluable role in ensuring food safety and quality control in the modern food industry.

## Figures and Tables

**Table 1:** Applications of non-destructive testing methods for estimating various egg quality parameters.

Technique	Parameters	Model	Accuracy of the Best Models Obtained for Each Algorithm	Notice (Spectrum Range)	Ref.
Near-infrared (NIR) spectroscopy	Indexes of weight loss rate, yolk index, and Haugh unit	LDA	91.40%	550–985 nm	[70]
	Integrated freshness index (IFI)	SVR	81.60%	500–900 nm	[71]
	Double yolk (DY) and single yolk (SY)	DA and SVM	96% and 100%	200–900 nm	[72]
	HU	SVMR and PLSR	87.00%	900–1700 nm	[73]
	Internal properties (Haugh unit, albumen height, yolk color, yolk weight, albumen weight, yolk coefficient (YC), and yolk height), and external quality (shell weight, strength, and thickness)	ANN	94.00%	400–1100 nm	[74]
	ACH, HU, albumen pH, TAH, and YC	ANN	100%		[75]
	Air chamber size, weight loss, and pH	PLS	91%		[52]
	Haugh units and pH of albumen	PLS	R^2^ = 0.82 and R^2^ = 0.86	200–1100 nm	[76]
Raman spectroscopy	Haugh unit, albumen pH, and air chamber diameter	PLSR	90.00%	100–3000 nm	[19]
	External and internal parameters of fake eggs	PLS-DA	100%	600–1800 nm	[77]
	Yolk freshness	PLS-DA	80%	950–3000 nm	[78]
Dielectric spectroscopy	Haugh unit, yolk index, yolk/albumen, and yolk weight	ANN	R^2^ = 0.998, R^2^ =0.998, R^2^ = 0.998, and R^2^ = 0.994, respectively	40 KHz–20 MHz	[79]
	Air cell, thick albumen height, and yolk index	ANN	R^2^ = 0.918, R^2^ = 0.854, and R^2^ = 0.912, respectively	3–20 GHz	[80]
Fluorescence spectroscopy	Vitamin A and FMRP	PCA-FDA	85.70% 63.90%		[81]
	Storage time	PCA-FDA	94.40%		[82]
Computer vision	Length, breadth, and volume	Classification model	99.88%, 98.26%, and 99.02%, respectively		[61]
	Haugh unit, yolk index, yolk/albumen ratio, and yolk weight	ANN	99.8%, 99.8%, 99.8%, and 99.4%, respectively		[79]
	Minimum, maximum, and effective radii, perimeter, and frontal area	Edge algorithm	95%		[83]
	Haugh unit using egg weight, long axis, and minor axis	MLR	86.50%		[84]
	Haugh unit and albumen pH	Levenberg–Marquardt	93% and 87%		[13]
	Dark spots on surface, soundness of eggshell	LFI (local fitting image)	92.50%		[85]
	Crack detection	New algorithm	94%		[86]
Hyperspectral imaging	HU	PLSR	91%	900–1700 nm	[87]
	HU	SVM, KNN, RF, NB, DAC, and LDA	100%, 88.75%, 95%, and 96.25%, respectively	400–2500 nm	[88]
	Freshness, bubble formation, or scattered yolk	SPA-SVM	R^2^_p_ = 0.87	350–1010 nm	[89]
Electronic noses and tongues	Textures, smells, and tastes	KNN, LDA, and SVM	96.70%		[90]
	Odor score, overall acceptability score, and HUs	FDA	R^2^ = 0.9441, R^2^ = 0.9511, R^2^ = 0.9725, and R^2^ = 0.9530, respectively		[91]
	Haugh unit and yolk index	Multiple linear regression (MLR) and backpropagation neural network (BPNN)	84%		[92]
	Shelf life of eggs	PLSR	95%		[93]

**Table 2:** Comparisons of the main features of non-destructive testing methods of egg freshness estimation.

Techniques	Spectral Information	Spatial Information	Multi-Constituent Information	Online Applications	Simplicity	Data Dimension	Speed of Analysis	Cost
NIR spectroscopy	√		√	√	√	1 D	Super rapid	Low
Raman spectroscopy	√		√	√	√	1 D	Rapid	Moderate
Dielectric spectroscopy	√		√	√	√	1 D	Rapid	Low
Fluorescence spectroscopy	√		√			1 D	Moderate	Moderate
Computer vision		√		√	√	2 D	Rapid	Low
Hyperspectral imaging	√	√	√			3 D	Moderate	High
Electronic noses						1 D	Slow	Moderate
Nuclear magnetic resonance						2 D	Slow	High

## Data Availability

No new data were created or analyzed in this study. Data sharing is not applicable to this article.
